# 

**Published:** 1894-04-07

**Authors:** 


PRICE TWOPENCE.

No. 393, Vol. XVI.—April 7th, 1894.

[Registered at the General Post Office as a Newspaper for transmission in the United Kingdom and Abroad.]

WITH EXTRA NURSING SUPPLEMENT.
Per Annum, 8s. 6d., post free.
Monthly Parts, 6d.; post free, 8d.
Ditto per Annum, 8s., post free.

